# Cold atmospheric plasma selectively induces G_0_/G_1_ cell cycle arrest and apoptosis in AR-independent prostate cancer cells

**DOI:** 10.7150/jca.54528

**Published:** 2021-08-17

**Authors:** Dong Hua, Dongyan Cai, Meng Ning, Lihui Yu, Zhifa Zhang, Peiyu Han, Xiaofeng Dai

**Affiliations:** 1 Wuxi School of Medicine, Jiangnan University, Wuxi 214122, China.; 2 Wuxi People's Hospital, Wuxi 214043, China.; 3 Affiliated Hospital of Jiangnan University, Wuxi 214000, China.; 4 School of Mechanical Engineering, Jiangnan University, Wuxi 214122, China.; 5 Jiangsu Key Laboratory of Advanced Food Manufacturing Equipment and Technology, Jiangnan University, Wuxi 214122, China.

**Keywords:** prostate cancer, cell apoptosis, G0/G1, cell cycle arrest, cold atmospheric plasma

## Abstract

**Purpose:** Androgen receptor-independent prostate cancers do not respond to androgen blockage therapies and suffer from high recurrence rate. We aim to contribute to the establishment of novel therapeutic approaches against such malignancies.

**Materials and Methods:** We examined whether and how cold atmospheric plasma delivers selectivity against AR-independent prostate cancers via cell viability, transwell assay, wound healing, cell apoptosis assay, flow cytometry, intracellular hydrogen peroxide determination assay, RONS scavenger assay and western blot using human normal epithelial prostatic cells PNT1A and AR-negative DU145 prostate cancer cells.

**Results:** We show that cold atmospheric plasma could selectively halt cell proliferation and migration in androgen receptor-independent cells as a result of induced cell apoptosis and G0/G1 stage cell cycle arrest, and such outcomes were achieved through modulations on the MAPK and NF-kB pathways in response to physical plasma induced intracellular redox level.

**Conclusion:** Our study reports cold atmospheric plasma induced reduction on the proliferation and migration of androgen receptor-independent prostate cancer cells that offers novel therapeutic insights on the treatment of such cancers, and provides the first evidence on physical plasma induced cell cycle G0/G1 stage arrest that warrants the exploration on the synergistic use of cold atmospheric plasma and drugs such as chemotherapies in eradicating such cancer cells.

## Introduction

Prostate cancer is the most common malignancy, excluding basal cell and squamous cell skin cancers, and ranks second for estimated cancer deaths among men in the United States 1. In recent years, the number of individuals receiving prostate cancer screening has gradually increased 2-4. Prostate cancers are androgen receptor (AR)-dependent in the early stage and can thus be treated effectively through androgen blockade. However, advanced prostate cancers are likely to be AR-independent and easily to recur, and therefore lack effective therapeutic strategies.

Cold atmospheric plasma (CAP) is an incompletely ionized type of physical plasma (the fourth state of matters). It contains reactive oxygen and nitrogen species with specific species being highly dependent on the gas used 5, 6. CAP has been widely applied in many bio-related fields such as bacterial disinfection, cell transfection, dentistry and wound healing 7-11, with its selectivity against cancer cells being firstly reported in 2007 12. Ever since, CAP was shown effective in treating multiple types of tumors such as breast 13, 14 and bladder cancers 15. Clinically, CAP was shown to be effective in partially alleviating superficial head and neck tumors and effectively reducing ulceration caused by cancers 16. The efficacy of CAP is dose-dependent, i.e., CAP could selectively induce cell cycle arrest, apoptosis and necrosis with the increase of CAP dose 17-19, and can lead to outcomes such as induced immunogenic cell death 20 and enhanced tumor sensitivity to chemical drugs 21.

Several studies have proposed and discussed the potential anti-cancer mechanism of CAP, with the most canonical one attributing the selectivity of CAP against cancer cells to the higher basal ROS level in cancer than normal cells 22. That is, with the elevated ROS level triggered by CAP, cells with higher basal redox level are more easily to reach the apoptotic threshold and thus undergo apoptosis 23. Later, the primary role of aquaporins in H2O2 transmembrane diffusion has been revealed, and the selectivity of CAP against cancer cells was attributed to the higher expression of aquaporins on cell cytoplasmic membranes that mediate the entry of more H_2_O_2_ (one kind of long-lived species in CAP) to cells 24. The most recent theory states that tumor progression requires the expression of membrane-bound catalase, and 1O2 (a type of short-lived species in CAP) can inactivate membrane-bound catalase and trigger the generation of tumor cell-derived secondary 1O2 and ROS-dependent apoptosis 25.

In this study, we examined whether and how CAP-activated medium (PAM) delivers selectivity against AR-independent prostate cancers using human normal epithelial prostatic cells PNT1A and AR-negative DU145 prostate cancer cells, respectively. It was reported that PAM could induce anti-proliferative effects in prostate cancer cells through redox and apoptotic signalling 26. Here, we found that PAM could selectively inhibit the proliferation, reduce the migration ability, induce the apoptosis of AR-independent prostate cells and, importantly, through blocking cancer cells at the G0/G1 cell cycle stage. This study provides the first evidence on PAM-induced cell cycle arrest in prostate cancer cells, and shed novel insights on therapeutic strategies against AR-negative prostate cancers through cell cycle blockage.

## Materials and methods

### Cell culture

The human normal epithelial prostate cell line PNT1A and prostate cancer cell line DU145 were obtained from Prof. YongQuan Chen's lab at Jiangnan University which were purchased from Chinese Academy of Sciences Cell Library. Both cell lines were cultured using RPMI1640 supplemented with 10% FBS and grown at 37 °C, 5% (v/v) CO_2_ in a humidified incubator.

### Plasma source and generation of plasma activated medium

**Figure 1:**
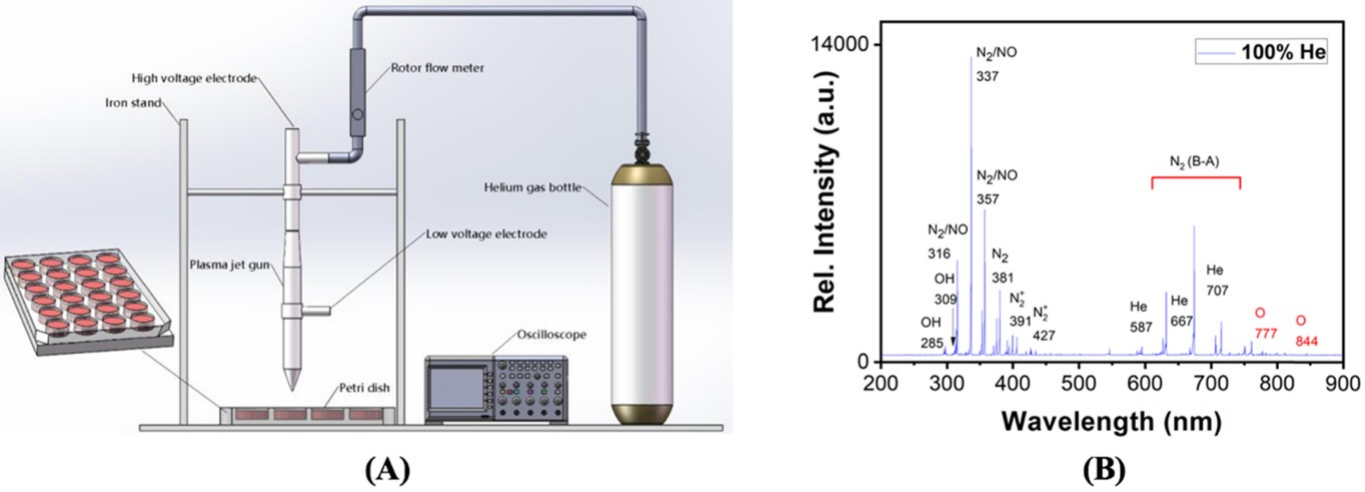
Schematic representation of the CAP device and experimental setup.

A custom-made plasma source was used to generate CAP here, which is a helium gas based plasma generator composed of a plasma jet gun, an iron stand, an oscilloscope, a rotor flow meter, and a helium gas bottle (Figure 1A). We applied a voltage of 1.2 KV on the high-voltage electrode, the gas flow rate was 1.2 L/min, the distance between the plasma jet gun and the surface of the culture medium was 1 cm, and the processing time was 2-5 min. The optical emission spectra (OES) of the gas phase CAP were detected using a spectrometer (Andor Shamrock SR-500i-A-R, England), where the optical probe was placed at a distance of approximately 10 mm from the center of the plasma source. PAM was prepared by allocating the medium in a 12-well plate with 2 mL/well before CAP exposure for 5 min, and was used to cultivate cells that were grown to 70-80% confluence immediately after preparation.

### Cell viability assay

Prostate cancer cells were plated in 96-well plates at a density of 1×10^4^ cells/well in 100 μL of complete culture medium and cultivated for 12-24 h until 60-70% confluence, followed by CAP treatment for 5 min. The first measurement was taken 8 h post CAP exposure. Cell viability was assessed by Cell Counting Kit-8 (Dojindo, Japan) according to the manufacturer's protocol. The absorbance was measured at 450 nm using EZ Read 800 microplate reader (Biochrom, UK).

### Transwell assay

Cell medium was added on the lower layer of 24-well culture plate and the chambers were placed in the medium. Cells with and without CAP exposure were incubated for 72 h followed by pancreatic digestion and re-suspension. Cells were added to the chambers, with 2×10^5^ cells per well. The culture media inside the chambers were discarded after 20 h, and cells were washed by phosphate buffered saline (PBS). Migrated cells under the chambers were fixed by methanol followed by staining using 0.1% crystal violet solution. The number of cells, regardless of cell size, from three randomly selected view fields were counted under microscope and used for data quantification.

### Wound-healing assay

Cells were plated in 6-well plates at a density of 1×10^6^ cells/well, and scratched by a p100 tip when cells attached to the bottom of the plate as a monolayer and reached the confluence of 90%. Cells in each well were incubated using CAP-activated serum-free medium for 24 h, 48 h and 72 h. Scratches were visualized using inverted phase contrast microscope and images were captured using a digital microscope camera. Images were captured for each well immediately after the medium was replaced with PAM, and after 8 h, 16 h and 24 h of incubation. The cell migration area was quantified by identifying the edges of the unfilled region and taking the integrated area using Image J software.

### Cell apoptosis assay

Apoptosis ratio was quantified using annexin V-FITC apoptosis detection kit (Dojindo, Japan). Cells were attached to a 6-well plate at a density of 1×10^5^ cells/well, and the medium was replaced by 2 mL PAM and incubated for 24 h when cell density reached 80% confluence. Cells were harvested and stained using annexin V and propidium iodide (PI). FACSCalibur (Becton Dickinson Biosciences, Franklin Lakes, NJ, USA) was used to calculate the apoptosis rate. Results were analyzed using FlowJo software (Ashland, OR).

### Cell cycle assay

Cells were grown in 6-well plates, PBS-washed and digested using EDTA-free trypsin. Cells were centrifuged using Eppendorf Centrifuge 5418 R at 1000 rpm for 5 min, and supernatant was removed to collect the pellets. Cell pellets were washed using 500 μL PBS, centrifuged at 1000 rpm for 5 min, and the supernatant was removed to retain the pellets. Cell pellets were re-suspended in 70% ethanol and placed in a 4 °C refrigerator overnight. Fixed cells were centrifuged at 1000 rpm for 5 min to remove the supernatant, and cell pellets were suspended in 500 μL PBS, supplemented with 5 μL Propidium iodide staining agent (purchased from Beyotime), and mixed on ice for 30 min. Cell cycle detection was performed using a BD Accuri C6 flow cytometer, and data analysis was performed using Flowjo software.

### Intracellular hydrogen peroxide determination assay

ROS fluorescent probe-DHE (dihydroethidium, sigma, United States) was used to measure intracellular superoxide anion that reflects the level of ROS in general. Data acquisition was performed under Nikon's fluorescence microscope following manufacture's protocol.

### RONS scavenger assay

Cells were cultured in RPMI1640 containing 10% (v/v) FBS in a 6-well plate, and placed in an incubator at 37 °C, 5% CO_2_ and saturated humidity untile 80 -90% confluence. 10 μL of sodium pyruvate (100 mM), uric acid (1 mM), mannitrol (2 M), Tiron (200 mM), hemoglobin (200 μM), monopotassium phosphate (10 mM) was added to 1 mL PAM, separately, before treating cells and performing cell flow cytometry.

### Western blot

The 6-well plates of the cultured cells were taken out, washed with PBS at 4 °C, and then lysed with RIPA protein lysate containing protease inhibitors and phosphatase inhibitors for 5 min on ice, followed by centrifugation at 12,000 rpm for 20 min at 4 °C. The supernatant was taken and the protein concentration was determined using a BCA protein assay kit. The same amount of protein (40 μg) was added to 10% sodium dodecyl sulfate-polyacrylamide gel electrophoresis (SDS-PAGE) and then transferred to the PVDF membrane. Non-specific binding sites were blocked for 1 h at the room temperature (RT) using 5% skim milk, followed by the PVDF membrane wash for 15 min, addition of primary antibody for 12 h and an appropriate horseradish peroxidase conjugated secondary antibody at RT for 1 h. Visualization of reactive protein bands was performed using High-sig ECL (enhanced chemiluminescence) western blotting substrate (Tanon). The expression level of reactive protein bands was quantified by taking the average integrated density using Image J software.

## Results

The optical emission spectra (OES) of gas phase plasma were enriched with hydroxide (OH), singleton oxygen (O), nitrogen oxides (NO) (Figure 1B). In the liquid phase, NO might become NO^2-^ and NO^3-^, OH might form H_2_O_2_, O might become O_3_, and O_3_/O might be further converted to O^2-^.

### PAM confers selectivity on prostate cancer cell proliferation and migration

**Figure 2:**
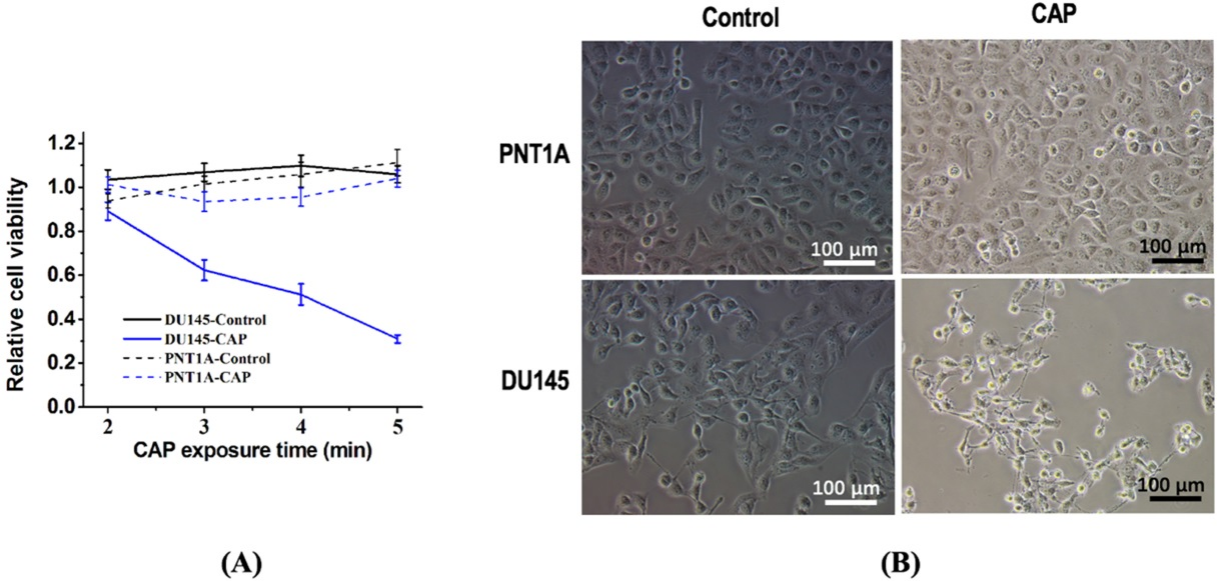
** CAP selectively reduces the viability of prostate cancer cells.** (A) Cell viability after CAP treatment at different time points. Treat the medium with 1.2 kV for 2, 3, 4, 5 min, and then measure the cell viability after 8 h of incubation. (B) Cell morphology after CAP exposure. PNTA1 and DU145 are normal prostate cells and prostate cancer cells respectively. CAP represents cold atmospheric plasma. Three independent replicates were performed. *p < 0.05 (Student's t-test).

The survival rate of DU145 prostate cancer cells significantly decreased (p=3.15E-3) in response to PAM treatment while that of normal prostate cells (PNT1A) did not considerably alter (Figure 2A). DU145 cells showed a more roundish morphology than PNT1A cells; the size and amount of DU145 cells were both shrinked on CAP exposure after 8 hours whereas those of PNT1A were not (Figure 2B).

**Figure 3:**
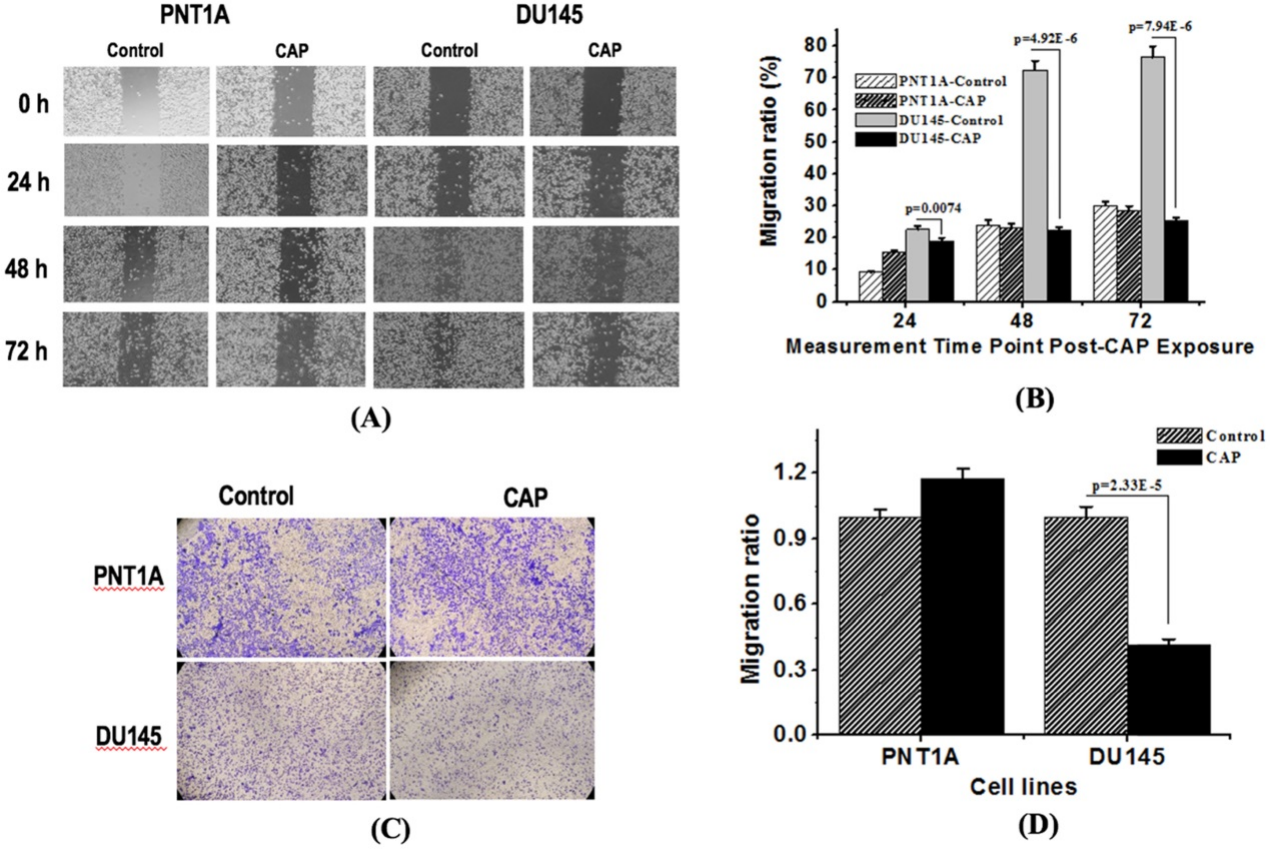
** CAP selectively halts the migration of prostate cancer cells.** (A) Transwell assay measuring the migration ability of PNT1A cells and DU145 cells before and 72 h after CAP treatment. (B) Quantification of fluorescence from the transwell assay. (C) Scratches of PNT1A and DU145 cells were photographed 24 h, 48 h and 72 h after CAP treatment. (D) Relative mobility of PNT1A and DU145 cells before and after CAP treatment. PNTA1 and DU145 are normal prostate cells and prostate cancer cells respectively. CAP represents cold atmospheric plasma. Three independent replicates were performed. *p < 0.05 (Student's t-test).

Both wound healing and transwell assays indicated the selective inhibition of PAM on the migration ability of prostate cancer cells. The migration rate of DU145 cells dropped from 78.6% to 20.7% at 48 h (p=4.92E-6) and from 81.9% to 23.4% (p=7.94E-6) at 72 h after PAM treatment from the wound healing assay (Figure 3A, 3B), and decreased 59.5% at 48 h after PAM treatment from the transwell assay (p=2.33E-5, Figure 3C, 3D), whereas significant decrease on the migration rate of PNT1A cells was not observed.

### PAM selectively triggers apoptosis in prostate cancer cells

**Figure 4:**
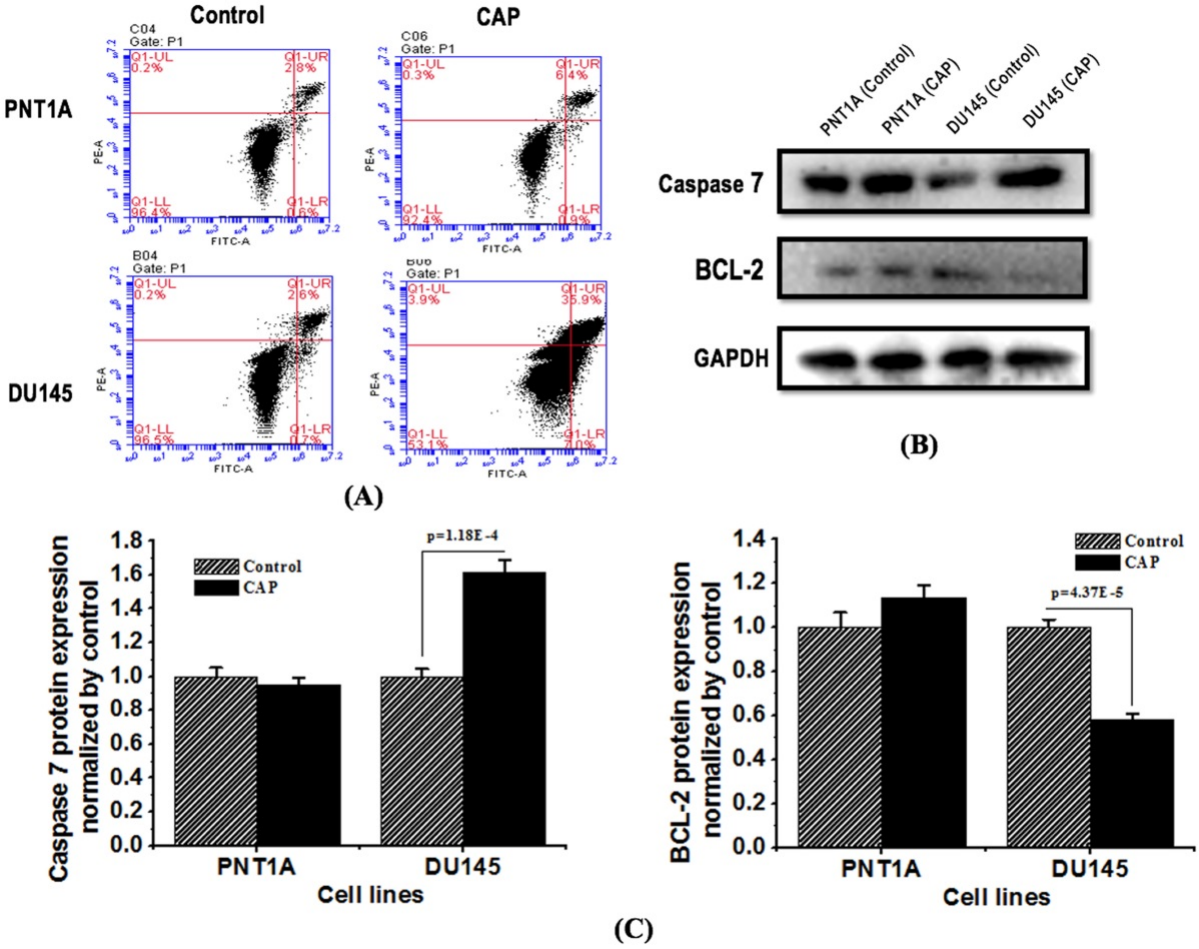
** CAP selectively triggers cell apoptosis in prostate cancer cells.** (A) Flow cytometry images showing cell apoptosis rate in response to CAP treatment. Treat the medium for 5 minutes, and then measure the apoptotic rate after 8 hours of incubation. (B) Expression of apoptosis related proteins caspase 7 and Bcl-2 after PAM treatment. (C) Standard quantification of the expression of apoptosis related proteins caspase 7 and Bcl-2. PNTA1 and DU145 are normal prostate cells and prostate cancer cells respectively. CAP represents cold atmospheric plasma. The medium was under CAP exposure for 5 minutes, and the protein was extracted after 8 hours of incubation. Three independent replicates were performed in western blot.

The apoptotic rate (including both early and late apoptosis) of DU145 cells increased from 7.3% to 42.9%, while that of PNT1A cells did not significantly alter (Figure 4A). The expression of apoptosis protein caspase 7 increased, and that of the anti-apoptotic protein Bcl-2 decreased, both with statistical significance (p=1.18E-4 for caspase 7, p=4.37E-5 for Bcl-2, Figure 4B, 4C), in DU145 cells in response to CAP treatment; yet those in PNT1A cells did not considerably alter.

### PAM selectively induces G0/G1 cell cycle arrest in prostate cancer cells

**Figure 5:**
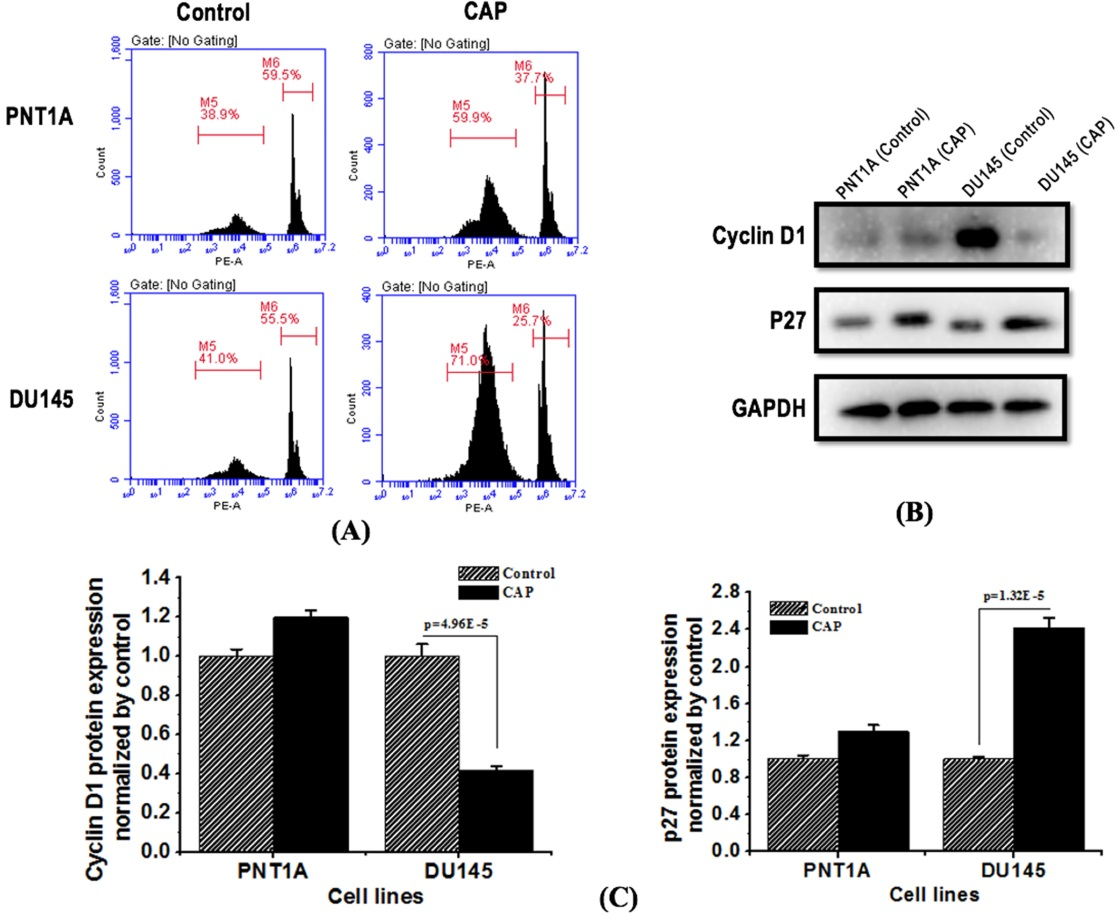
** CAP selectively induces G0/G1 cell cycle arrest in prostate cancer cells and inhibits prostate cancer activity.** (A) Flow cytometry images showing cells' quantity distribution at the G0/G1, S, G2/M phase in response to CAP treatment. (B) Expression of cell cycle related proteins cyclin D1 and p27 in PNT1A cells and DU145 cells after PAM treatment. (C) Standard quantification of the expression of cell cycle related proteins cyclin D1 and p27. PNTA1 and DU145 are normal prostate cells and prostate cancer cells respectively. CAP represents cold atmospheric plasma. The medium was under CAP exposure for 5 minutes, and the protein was extracted after 8 hours of incubation. Three independent replicates were performed in western blot.

Flow cytometry showed that PAM treatment significantly induced prostate cancer cell cycle arrest at the G0/G1 phase. The proportion of cells arrested at the G0/G1 phase increased from 41.0% to 71.0% and that arrested at the G2/M phase decreased from 55.5% to 25.7% in DU145 cells; in contrast, much less alteration on cell cycle was observed in PNT1A cells (Figure 5A). Western blot showed that PAM treatment significantly reduced the expression of cycle-related protein cyclin D1 (p=4.96E-5, Figure 5B, 5C), and increased the expression of cyclin-dependent kinase inhibition p27 protein with statistical significance (p=1.32E-5, Figure 5B, 5C).

### PAM selectively increases the intracellular ROS level of prostate cancer cells

**Figure 6:**
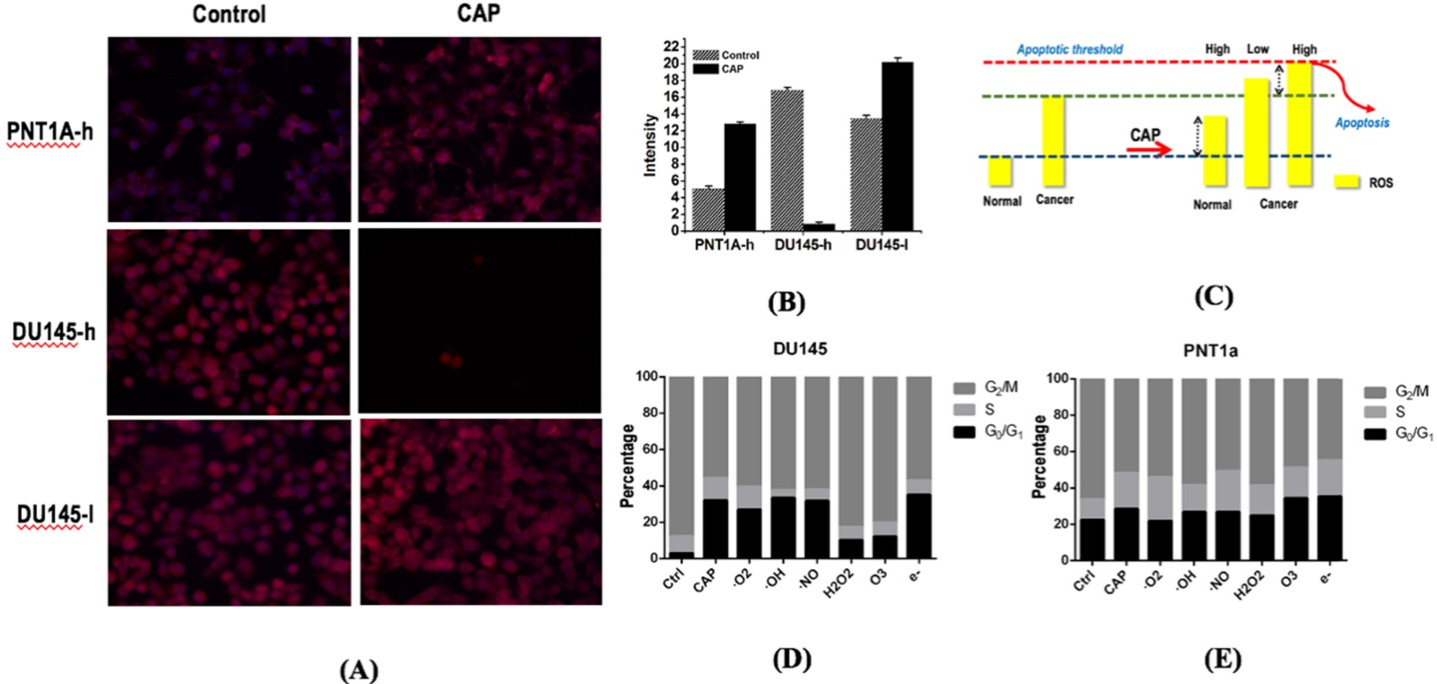
** Alterations of intracellular ROS levels in PNT1A cells and DU145 cells receiving different CAP doses.** (A) Intracellular ROS levels and (B) quantification in PNT1A and DU145 cells under different CAP doses (high dose is 1.2 kV and 5 minutes of CAP treatment; low dose is 1.0kV and 3minutes of CAP treatment). (C) Representative scheme showing ROS-associated CAP selectivity on prostate cancer cells. PNTA1 and DU145 are normal prostate cells and prostate cancer cells respectively. Percentage of G_0_/G_1_ stage after removing each RONS component in (D) prostate cancer cells DU145 and (E) normal cells PNT1A. Hydroxyl radical (OH**·**), hydrogen peroxide (H_2_O_2_), ozone (O_3_), superoxide anion (O^2·-^), nitric oxide (NO·), and electron (e^-^) are quenched by mannitrol, uric acid, tiron, hemoglobin and monopotassium phosphate, respectively. CAP represents cold atmospheric plasma. Three independent replicates were performed.

Immunofluorescence detection assay showed that PAM treatment could significantly increase cellular ROS levels that ultimately lead to cancer cell apoptosis. DU145 cells showed higher ROS level than PNT1A cells (p=7.52E-3, Figure 6A, 6B). The basal ROS level of DU145 cells was higher than that of PNT1A cells, and the ROS levels of DU145 and PNT1A cells both increased on CAP exposure (Figure 6A, 6B). The cellular ROS level significantly dropped under high dose CAP exposure (Figure 6A, 6B) in DU145 cells due to likely triggered cell apoptosis. While cancerous cells have higher basal ROS level than normal cells, they could be able to more easily reach the apoptotic threshold and undergo cell death 23, 27 that lead to dramatically reduced ROS intensity (Figure 6C).

### H_2_O_2_ and O_3_ are the leading RONS that trigger G_0_/G_1_ cell cycle arrest

CAP could selectively increase the G_0_/G_1_ stage of prostate cancer cells DU145 as compared with normal cells PNT1A (Figure 6D, 6E). By quenching each reactive species in CAP, H_2_O_2_ and O_3_ were found capable of substantially lowering the increased G_0_/G_1_ proportion triggered by CAP (Figure 6D, 6E), suggestive of the primary contribution of these two components in triggering the G_0_/G_1_ cell cycle arrest in prostate cancer cells DU145.

### CAP selectively suppresses phosphorylation of key proteins involved in MAPK and NF-kB pathways

**Figure 7:**
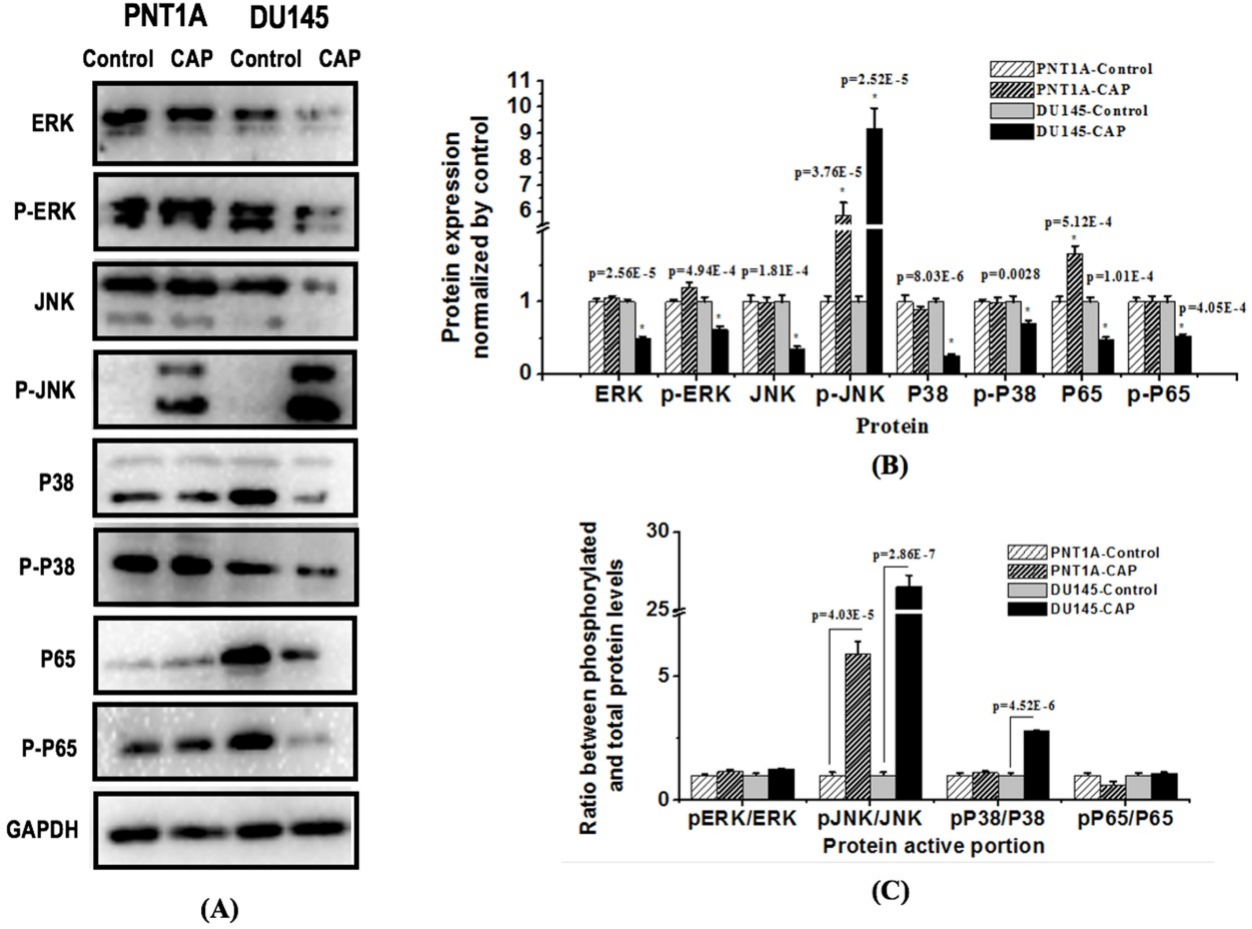
** Western blotting showing the effect of CAP on key proteins in MAPK and NF-kB pathways in prostate cancer cells.** (A) Expression and (B) quantification of key proteins involved in MAPK and NF-kB pathways. PNTA1 and DU145 are normal prostate cells and prostate cancer cells respectively. CAP represents cold atmospheric plasma. Three independent replicates were performed in western blot.

In order to further explore the mechanism of CAP-induced cancer cell death, we examined the total level and phosphorylation status of c-Jun N-terminal kinase (JNK), extracellular signal regulated kinase (ERK), and p38, each representing one MAPK pathway, as well as p65, the most common form of NF-Kb, in PNT1A and DU145 cells after PAM treatment for 8 h (Figure 7). The results showed that PAM could significantly inhibit the amount and activity of ERK (p=2.56E-5 for total protein, p=4.94E-4 for ERK phosphorylation), p38 (p=8.03E-6 for total protein, p=0.0028 for p38 phosphorylation) and p65 (p=5.12E-4 for total protein, p=1.01E-4 for p65 phosphorylation) in prostate cancer cells; the phosphorylation level of JNK was significantly increased (p=2.52E-5) while its total protein level was reduced with statistical significance (p=1.81E-4) in DU145 cancer cells. The relative phosphorylation levels of JNK and p38 as compared with the control were significantly increased in DU145 cells (p=2.86E-7 for JNK, p=4.52E-6 for p38, Figure 7C), and that of ERK and p65 did not vary with statistical significance.

## Discussion

We found from our study that PAM could selectively halt prostate cancer cell proliferation and migration. PAM was found to convey selectivity in multiple types of cancer cells including, e.g., melanoma 12, breast cancer 13, 14 and head and neck carcinomas 16. However, relatively little was reported on the efficacy of PAM in killing prostate cancer cells. Previously, it was reported that PAM could induce the anti-proliferative effect in prostate cancer cells LNCaP and PC-3 26, 28. Here, in this study, we demonstrated that PAM conferred both anti-proliferative and anti-migrative abilities in AR-independent prostate cancer cells DU145 without significantly affecting normal prostate cells; and reported that halted proliferation and migration in response to PAM treatment might be attributable to apoptosis and G0/G1 cell cycle arrest that were specifically triggered in prostate cancer cells after PAM treatment.

The cell scratch and transwell assays were conducted to evaluate cell invasion and migration abilities. It was shown that human normal epithelial prostatic cells PNT1A seemed to have a higher migrative ability but a significantly lower invasive potential than AR-negative prostate cancer DU145 cells (Figure 3A, 3C). As migration refers to normal cell movement, yet invasion describes cells' ability in invading the surrounding tissue, we could arrive at the conclusion that though malignant DU145 cells have a lower migrative ability, they are more invasive than the normal PNT1A cells, and PAM could effectively halt their invasive and migrative abilities. The seemingly more migrative feature of PNT1A cells than DU145 cells may also be resulted from the size difference of both cell types as cells were counted regardless of cell size. The use of normal epithelial prostatic cells as the control in assessing the efficacy of PAM against AR-negative prostate cancer cell migration is to remove the effect of cell proliferation on cell invasion in the cell scratch assay.

The simultaneously induced G0/G1 cell cycle arrest and cell apoptosis in response to PAM treatment were due to enhanced intracellular ROS levels. Malignant cells typically have higher basal ROS levels than normal cells and thus are easier to reach the apoptotic threshold (Figure 6D); appropriate dosage control of CAP can selectively trigger the cell death program in cancer cells due to the increased cellular ROS 29 and arrest a fraction of cancer cells at the G0/G1 stage. This is clinically relevant as cells under cell cycle arrest on PAM treatment may restore the activity once PAM is removed. Yet, combinatorial use of PAM with other therapeutic strategies capable of transiting 'dormant' cells to the active state or leading them to apoptosis is recommended in practice to eradicate androgen receptor-independent prostate cancer cells.

Two canonical pathways, i.e., MAPK and NF-kB signaling, were found to be affected and contribute to PAM-triggered cell cycle arrest and apoptosis in prostate cancer cells. It was reported that PAM could effectively reduce the phosphorylation level of NF-kB and JNK but had little effect on ERK and p38 in triple negative breast cancer cells 13; and could activate p38 and JNK signaling in melanoma cells 30. Here, we found that NF-kB, ERK and p38 phosphorylation levels were significantly reduced, and that of JNK was increased in prostate cancer cells on CAP exposure. These collectively suggest the potential differential use of signaling molecules in different types of cancer cells that may be driven by their different genetic backgrounds and imply the importance of interactions between CAP particles and cell intrinsic molecular background in the choice of signaling that mediate PAM efficacy.

Though we demonstrated the efficacy and potential anti-cancer mechanism of PAM in prostate cancer cells, there exist several limitations that need further explorations. First, lack of animal experiments.* In vivo* assays using cell line inoculated mouse or patient-derived tumor xenograft (PDX) models need to be conducted to confirm our *in vitro* findings. Second, limited number of cell lines. We only used one prostate cancer and one normal prostate cell line in this study which prohibits us from exploring the potential differential efficacies of PAM on prostate cancer cell lines harboring different genetic background; and this may ultimately limit us from deciphering the anti-cancer mechanism of PAM from a more general level. Third, limited pathways being explored. We only explored the potential contribution of two canonical cancer associated pathways, i.e., MAPK and NF-kB signaling, on PAM-induced cancer cell response in this study according to 13. Other pathways such as Wnt and TGF-beta may also contribute to this process or orchestrate with MAPK and NF-kB signaling to collectively tilt the behavior of cancer cells towards a less malignant stage. Lastly, we may test whether our conclusions hold in other type of cancers which worth further investigations.

## Conclusion

We conclude from this study that PAM could selectively halt prostate cancer cell proliferation and migration through G0/G1 cell cycle arrest and apoptosis in response to increased cellular ROS levels, and such efficacies were selective on cancer cells without affecting normal prostate cells. In addition, such PAM-mediated anti-cancer effects are mediated via reduced phosphorylation level of key proteins involved in MAPK and NF-kB pathways. We are the first to explore and report cell cycle arrest triggered by PAM on prostate cancer cells. This study not only contributes to our understandings on PAM selectivity against cancer cells and its potential mechanism, but also warrants studies on synergies between PAM and chemotherapies that may potentially rewire cell cycle arrest towards apoptosis in that particular cell cohort if translated into clinics.

## Figures and Tables

**Figure 8:**
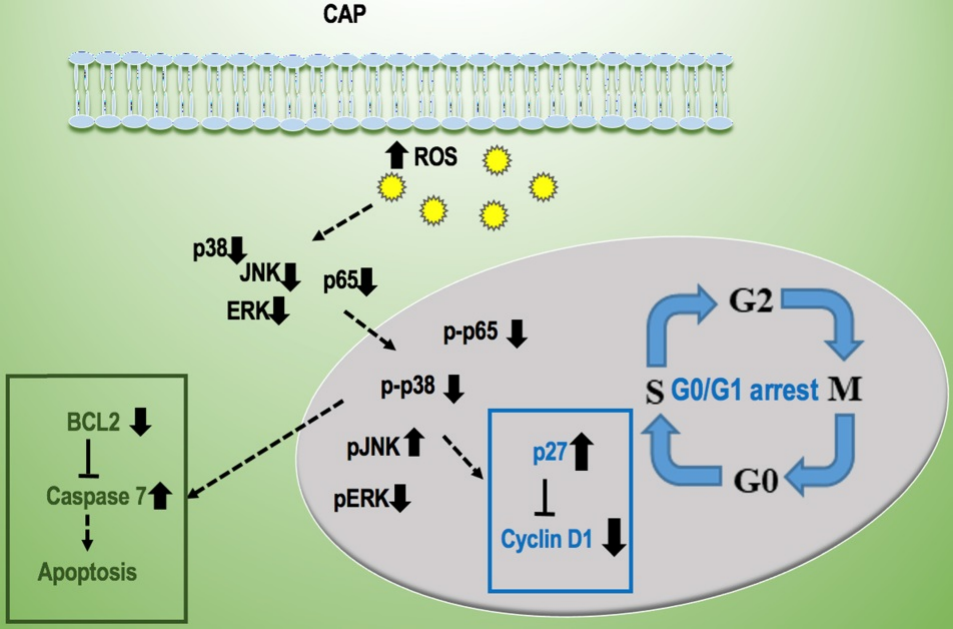
Conceptual network showing the regulatory mechanism of CAP on prostate cancer cells.
